# The effect of aqueous extract of orchid root on the structure of ovary and hypothalamic-pituitary-gonadal hormones in polycystic ovary syndrome rat model: An experimental study

**DOI:** 10.18502/ijrm.v22i3.16164

**Published:** 2024-05-15

**Authors:** Hassanali Abedi, Armin Zarrin-Mehr, Bahareh Ebrahimi, Hoda Haghshenas, Negar Parvin, Hossein Kargar Jahromi

**Affiliations:** ^1^Research Center for Noncommunicable Disease, Jahrom University of Medical Sciences, Jahrom, Iran.; ^2^Student Research Committee, Jahrom University of Medical Sciences, Jahrom, Iran.; ^3^Shiraz Geriatric Research Center, Shiraz University of Medical Sciences, Shiraz, Iran.

**Keywords:** PCOS, Orchid, Ovary, GnRH, LH, FSH.

## Abstract

**Background:**

Some medical conditions, including polycystic ovarian syndrome (PCOs), may lead to infertility. In PCOs, hormonal imbalance is significant. Antioxidants such as natural antioxidants have many health benefits, including positive effects on hormone production.

**Objective:**

Since herbal medicines are more acceptable to people, the present study was designed to evaluate the effect of an aqueous extract of orchid (SA), with antioxidative effects, on the structure of the ovary and the hypothalamic-pituitary-gonadal axis hormones and free testosterone in PCOs rats.

**Materials and Methods:**

In this experimental study, 64 healthy female Wistar rats (180–200 gr) were randomly divided into 60 and 89 day control groups, PCOs, and 4 PCOs + SA groups that received 40, 80, 160, and 320 mg/kg of SA. Serum levels of gonadotropin-releasing hormone, estrogen, progesterone, testosterone, follicle-stimulating hormone, and luteinizing hormone were measured. In addition, the ovaries were extracted and examined histologically.

**Results:**

The amount of primordial, primary, secondary, and Graafian follicles and serum levels of follicle-stimulating hormone and progesterone hormones decreased in PCOs groups, while atretic follicles and the serum levels of gonadotropin-releasing hormone, luteinizing hormone, estrogen, and free testosterone were increased. SA at different doses regulated hormonal and histological imbalances caused by PCOs, and 320 mg/kg was the most effective.

**Conclusion:**

The aqueous extract of orchids root can have a positive effect on the improvement of polycystic ovary syndrome. This effect can be achieved by regulating the level of sex hormones and correcting follicular abnormalities in the ovarian tissue.

## 1. Introduction

Polycystic ovary syndrome (PCOs) is one of the most important causes of infertility; morphological manifestations of polycystic ovaries are determined by pelvic ultrasound, and on the other hand, a range of symptoms such as obesity, hyperandrogenism, menstrual disorders, infertility, and even heart disease and stroke can occur individually or in combination (1–3). PCOS is associated with abnormal secretion of gonadotropins, increased production of steroids in the ovaries, and sometimes insulin resistance (4).

Although there is no cure for PCOs, there are many ways to manage its symptoms, using lifestyle changes and medication (if needed) (5). Today, various drugs are used to treat polycystic ovaries, each of which has several side effects. Chronic management of PCOs, including long-term use of chemical drugs, is not recommended (6). PCOs is associated with increased levels of inflammation (tumor necrosis factor, interleukin 6, and interleukin 18) and markers of oxidative stress (malondialdehyde, total antioxidant capacity, nitric oxide, and glutathione) (7, 8). For example, in a case-control study of 100 Nigerian women, it was found that serum malondialdehyde was significantly higher in PCOs women (n = 50) compared to controls (n = 50) and total antioxidant capacity was significantly lower (8).

Traditional medicine practitioners prescribe herbal medicines due to their low risks and side effects. One such plant, widely consumed in India, Nepal, China, and Europe, is Orchis, lancibracteata (K. koch) Renz Dactylorhiza, formerly known as *Orchis mascula* L., belonging to the orchid family. This plant contains nitrogenous substances, hydroxybenzaldehyde, ferulic acid, quercetin, daucosterol, cirsilineol, steroids, and glucomannan (9, 10). In traditional medicine, this plant was used as a sexual enhancer and erectile dysfunction treatment; it also increases physical strength and energy.

The orchid also increases the number of Leydig cells, which secrete testosterone in response to luteinizing hormone (LH) (11). An increase in testosterone can release dopaminergic chemical mediators in the brain, and it has been shown that there is a significant relationship between the release of dopamine in the nucleus accumbens and the improvement of sexual activity (11, 12). Studies have shown that the aqueous extract of orchid root (SA) can stimulate oogenesis and increase the hormones LH, progesterone, follicle-stimulating hormone (FSH), and estrogen (13).

Considering the antioxidant properties of orchids and the definite role of oxidative stress in PCOs (14, 15), this study was conducted with the aim of investigating the effect of SA on the treatment of polycystic ovary in adult female Wistar rats.

## 2. Materials and Methods

### Developing polycystic ovaries in rats

To induce PCOs, estradiol valerate (4 mg/kg, Osweh Company, Iran) was dissolved in 0.2 mg sesame oil and injected once intramuscularly and once in the back of the thigh on the abdominal surface. Estradiol valerate causes irregular cycles and anovulation in adult rats. After 60 days, polycystic ovarian disease was developed due to ovarian characteristics similar to PCOs, large number of atretic follicles and cysts (16, 17).

### Preparing orchid extract

Pour 100 gr of the fresh and chopped SA into 500 cc of ethanol (96%) and place the resulting mixture in a rotodoxia device at room temperature for 24 hr until a uniform mixture is obtained. The filtered solution was centrifuged at a speed of 3000 rpm for 5 min and the resulting solution was placed in ambient conditions for 48 hr to become a solid, dry, and alcohol-free extract. The obtained dry extract was dissolved in distilled water for use (11, 18).

### Experimental animals

For this experimental study, 64 female Wistar rats (180–200 gr, 9–10 wk old) were used. For accumulation, animals were kept in the animal room (23 ± 2 C) for 1 wk. 12 hr of light/dark cycle, and ambient humidity of 50–55% were observed. Vaginal smears were initially taken from all rats; animals in the estrous phase were selected, weighed, and kept in their cages (each cage contained 4 rats). Animals had access to adequate food and water.

### Test design

The animals were randomly divided into 8 groups (n = 8/each). To adapt animals before intervention, they were kept in the Jahrom University of Medical Sciences animal laboratory for a week. The environment dark and light cycle and the humidity were at standard rate, 12/12 hr and 50–55%, respectively. According to previous articles, the prescribed concentration was 40, 80, 160, and 320 mg/kg of body weight (19, 20). Therefore, the control and experimental groups in this study include healthy control groups 1 and 2: without receiving any substance and under normal conditions for 60 and 89 days, respectively. PCOs 1 and 2: after intramuscular injection of estradiol valerate, ovarian cyst formation is maintained for 60 and 89 days. Experimental groups (PCOs + SA [1–4]): after the formation of ovarian cyst (after 60 days), daily doses of 40, 80, 160, and 320 mg/kg SA were administrated intraperitoneally by insulin syringe at 10:00 AM daily for 28 days, and according to the body weight.

### How to draw blood and check hormones

At the end of the study, after weighing the animals, blood samples were taken directly from the hearts of animals using a 5 cc syringe (under anesthesia with ketamine [100 mg/kg] and xylazin [20 mg/kg]), and their serum was collected by centrifugation (for 15 min at 3000 rpm). It was then stored in a freezer -20 C until further usage. To measure the hormones, such as gonadotropin-releasing hormone (GnRH), FSH, LH, estrogen, progesterone, and free testosterone, enzyme-linked immunosorbent assay kits for rats made by Crystal Day China were used (Cat. No.: GnRH: E0186Ra, LH: E0179Ra, FSH: E0182Ra, testosterone: E0903Ra, estradiol: E0174Ra, progesterone: E0741Ra).

### Histological examination

After 28 days, ovarian tissue (left and right) was rapidly isolated using the standard method of anesthetized animals. After weighing with a digital scale with an accuracy of 0.001, it was placed in a container containing formalin 5%. After 48 hr, the ovarian tissue was removed from the solution and the normal stages of tissue passage were performed, then paraffin slices with a thickness of 5 µ were prepared as serial sections. 10 sections of each ovary were selected and stained with the hematoxylin and eosin staining method, and the initial, primary, secondary, graph, and atresia follicles were studied by light microscopy.

### Ethical considerations

In this study, all ethical issues regarding how to work with laboratory animals have been considered by the Jahrom University of Medical Sciences, Jahrom, Iran (Code: IR.JUMS.REC.1394.092).

### Statistical analysis

At first, the normality of the data were measured using the Shapiro-Wilk normality test. Data were analyzed by Duncan test using SPSS software version 20 (SPSS, Chicago, IL, USA), and a significance level of 0.05 was considered. All data were reported as mean ± SD.

## 3. Results

This study showed that using SA causes changes in sex hormones and ovarian tissue in rats with PCOs.

### Biochemical studies

In PCOs groups (PCOs 60 and 80 days), a significant increase in the serum levels of LH and estrogen and a significant decrease in progesterone and FSH were observed compared to other groups (p < 0.001). In all the PCOs groups, a significant increase in the serum free testosterone and GnRH level was observed compared to control groups (p < 0.001). Also, in the PCOs + SA 320 mg/kg group, a significant decrease in the serum free testosterone and GnRH level was detected compared to all of PCOs groups (p < 0.001). PCOs groups receiving SA (doses of 80, 160, and 320 mg/kg) significantly increased serum progesterone level (PCOs 60 days: p = 0.001, p < 0.001; PCOs 80 days: p < 0.001), and significantly decreased free testosterone level (PCOs 60 days: p = 0.017, p < 0.001; PCOs 80 days: p = 0.007, p < 0.001) compared with the PCOs groups (PCOs 60 and 80 days). PCOs groups receiving SA (doses of 40, 80, 160, and 320 mg/kg) significantly increased serum FSH level (PCOs 60 days: p = 0.01, p < 0.001; PCOs 80 days: p = 0.003, p < 0.001), and significantly decreased LH (PCOs 60 days: p = 0.025, p < 0.001; PCOs 80 days: p = 0.002, p < 0.001), and estrogen (p < 0.001) compared with the PCOs groups (PCOs 60 and 80 days).

**Figure 1:**
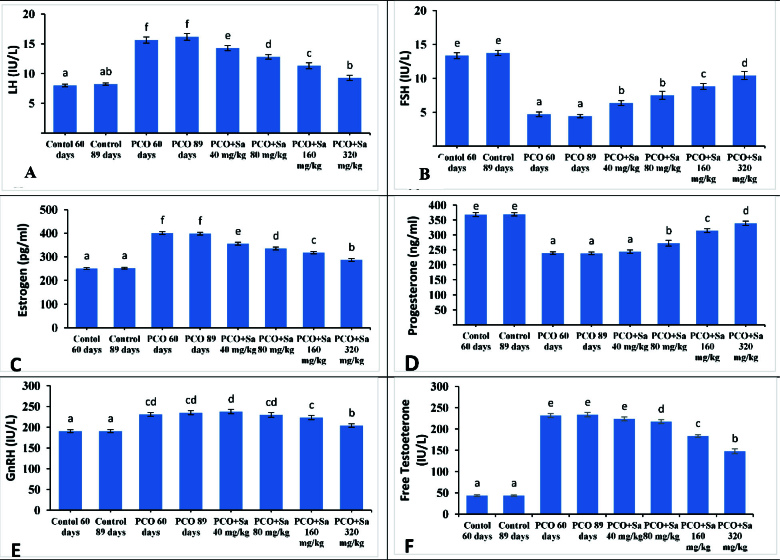
Comparison of mean LH, FSH, estrogen, progesterone, GnRH, and free testosterone concentrations in different groups based on the Duncan test. Each group means that at least one shared letter has no significant difference. P < 0.05 is considered statistically significant. Each group means that at least one shared letter has no significant difference. P < 0.05 is considered statistically significant. Gonadotropin hormone-releasing hormone (GnRH), follicle-stimulating hormone (FSH), luteinizing hormone (LH), polycystic ovary syndrome (PCOs), aqueous extract of orchid root (SA).

Serum LH level in the PCOs + SA 320 mg/kg group did not change significantly compared to the PCOs2 group (PCOs 80 days: p = 0.08). The dose of 320 mg/kg of SA showed the greatest effect compared to other doses in all variables (Figure 1).

### Histopathological examinations

The mean number of primary follicles in healthy groups was significantly higher than in other groups. In all studied groups, a significant decrease in primordial, primary, secondary, and graph follicles and a significant increase in atresia follicles were observed. Primordial follicles were significantly higher in 160 and 320 mg/kg SA than PCOs groups and the PCOs + SA 40 mg/kg group. No significant difference was observed in the mean number of primary follicles between the PCOs groups and the PCOs + 80 mg/kg SA group.

In addition, a significant difference was observed between the mean number of primary follicles in experimental groups PCOs + 80, 160, and 320 mg/kg SA. However, the experimental groups PCOs + 160 and 320 mg/kg SA had significantly more primary follicles than the PCOs groups. In the PCOs + SA 80, 160, and 320 mg/kg groups, the number of secondary follicles and graph follicles showed a significant increase compared to the PCOs groups, and the number of primary follicles only in the groups PCOs + SA 160 and 320 mg/kg showed a significant increase compared to the PCOs group.

In all PCOs groups receiving SA (all 4 studied doses), the number of atresia follicles showed a significant decrease compared to the PCOs groups. The dose of 320 mg/kg SA showed the greatest effect on primary, secondary, graph, and atresia follicles compared to other doses (Table I).

**Table 1:** The effect of different doses of aqueous extract of orchid root on PCOs Wistar rats follicles


**Groups**	**Healthy control group 1**	**Healthy control group 2**	**PCOs1**	**PCOs2**	**PCOs + SA 40 mg/kg**	**PCOs + SA 80 mg/kg**	**PCOs + SA 160 mg/kg**	**PCOs + SA 320 mg/kg**	**P-value (between groups)**
**Atresia follicles**	0.00 ± 0.00 (0 [0–0]) ${\;}^{\text{a}}$	0.00 ± 0.00 (0 [0–0]) ${\;}^{\text{a}}$	6.12 ± 1.24 (6 [7–5.5]) ${\;}^{\text{e}}$	6.12 ± 0.99 (6 [6.5–5.5]) ${\;}^{\text{e}}$	5.00 ± 1.51 (4.5 [6.5–4]) ${\;}^{\text{d}}$	3.87 ± 1.12 (4 [5–3]) ${\;}^{\text{c}}$	3.25 ± 1.03 (3 [4–2.5]) ${\;}^{\text{c}}$	1.12 ± 0.83 (1 [2–0.5]) ${\;}^{\text{b}}$	< 0.001
**Graph follicles**	5.37 ± 1.06 (5.5 [6–4.5]) ${\;}^{\text{c}}$	5.50 ± 1.19 (5.5 [6.5–4.5]) ${\;}^{\text{c}}$	0.12 ± 0.03 (0 [0–0]) ${\;}^{\text{a}}$	0.00 ± 0.00 (0 [0–0]) ${\;}^{\text{a}}$	0.37 ± 0.07 (0 [0.5–0]) ${\;}^{\text{a}}$	1.25 ± 1.16 (1.5 [2–0]) ${\;}^{\text{b}}$	1.75 ± 1.16 (2 [2.5–1]) ${\;}^{\text{b}}$	2.12 ± 1.80 (3 [3.5–1]) ${\;}^{\text{b}}$	< 0.001
**Secondary follicles**	5.37 ± 1.30 (5 [6–4.5]) ${\;}^{\text{c}}$	5.37 ± 1.06 (5 [6–4.5]) ${\;}^{\text{c}}$	0.50 ± 0.07 (0 [1–0]) ${\;}^{\text{a}}$	0.25 ± 0.04 (0 [0.5–0]) ${\;}^{\text{a}}$	0.87 ± 0.08 (1 [1.5–0]) ${\;}^{\text{a}}$	2.50 ± 1.06 (2 [3.5–2]) ${\;}^{\text{b}}$	3.00 ± 1.06 (3 [4–2.5]) ${\;}^{\text{b}}$	3.50 ± 1.06 (4 [4–2.5]) ${\;}^{\text{b}}$	< 0.001
**Primary follicles**	5.37 ± 1.30 (6 [6–4.5]) ${\;}^{\text{d}}$	5.50 ± 1.41 (5 [6.5–4.5]) ${\;}^{\text{d}}$	2.12 ± 1.12 (2 [3–1]) ${\;}^{\text{ab}}$	1.37 ± 0.51 (1 [2–1]) ${\;}^{\text{a}}$	1.62 ± 0.74 (1.5 [2–1]) ${\;}^{\text{a}}$	2.25 ± 0.70 (2 [3–2]) ${\;}^{\text{b}}$	2.87 ± 0.99 (3 [3.5–2.5]) ${\;}^{\text{bc}}$	3.62 ± 1.50 (4 [5–2.5]) ${\;}^{\text{c}}$	< 0.001
**Primordial follicles**	6.37 ± 1.06 (6.5 [7–5.5]) ${\;}^{\text{c}}$	6.50 ± 1.06 (6 [7.5–6]) ${\;}^{\text{c}}$	3.25 ± 0.70 (3 [4–3]) ${\;}^{\text{a}}$	3.25 ± 0.88 (3 [3.5–3]) ${\;}^{\text{a}}$	3.12 ± 0.83 (3 [4–2.5]) ${\;}^{\text{a}}$	4.00 ± 1.30 (4 [5–3]) ${\;}^{\text{b}}$	4.50 ± 1.41 (5 [5.30–3.5]) ${\;}^{\text{b}}$	4.62 ± 1.50 (5 [6–3.5]) ${\;}^{\text{ab}}$	< 0.001
Data presented as Mean ± SD (MD, IQR), Duncan test. Each group means that at least one shared letter has no significant difference. P < 0.05 is considered statistically significant. Each group means that at least one shared letter has no significant difference. P < 0.05 is considered statistically significant. Polycystic ovary syndrome (PCOs), aqueous extract of orchid root (SA)									

## 4. Discussion

The results of the present study indicated that in animals with PCOs, the use of aqueous extract of SA in a dose-dependent manner causes a change in the hormonal levels as well as the histological appearance of the ovaries. These changes included an increase in the initial, primary, secondary, and graph follicles, and a decrease in atresia follicles. Regarding hormones, it increases the serum levels of progesterone, GnRH, and FSH while decreasing the level of estrogen, free testosterone, and LH. Recent research has shown that herbal extracts have measurable benefits on PCOs symptoms and the development of new treatments (21, 22).

Current research has confirmed previous studies. Testosterone was found to be increased, whereas LH and FSH decreased during 60 or 89 days in PCOs rats. In addition, a significant decrease was observed in progesterone compared to healthy animals. The use of SA could partially compensate for changes in LH, FSH, progesterone, etc. In PCOs, serum LH levels increase, followed by increased estrogen levels, ovulation, and disruption of the normal menstrual cycle. At the same time, SA reduces non-ovarian levels in a dose-dependent manner. SA (320 mg/kg) normalized serum LH levels due to the 3 substances in SA, quercetin, daucosterol, and cercilinol, which have estrogenic potency (23, 24). Estrogen effectively inhibits gamma-aminobutyric acid neurons in preoptic regions on LH. These neurons reduce LH through negative feedback. In other words, if gamma-aminobutyric acid neurons are inhibited, an LH surge can be expected (25, 26). Therefore, this hormone increases in the presence of estrogen and inhibition of gamma-aminobutyric acid neurons. Also, SA increases the serum concentration of leptin hormone and reduces food intake in rats (27). Leptin is one of the most critical controllers in the release of LH from the pituitary gland. This hormone affects the secretion of lutein by increasing nitric oxide in the pituitary and hypothalamus (28). Therefore, increasing leptin hormone is another possible mechanism for increased LH release due to SA. Studies have shown that insulin and the insulin-like growth factor-1 stimulate progesterone synthesis in ovarian luteal cells (29). Ferulic acid and quercetin in SA effectively increase the concentration of insulin and insulin-like growth factor-1 (30, 31). These compounds can be effective in stimulating progesterone synthesis. Despite the large amounts of asteroids in the orchid plant and the presence of the cytochrome P450 enzyme in one of its compounds called quercetin, this enzyme is probably able to convert large amounts of cholesterol into progesterone, thereby causing a significant increase in progesterone (32).

No difference was observed in the number of primary follicles in PCOs compared to healthy individuals. Normal and above normal, LH had no effect on primordial follicles. This finding contradicts with previous studies, and therefore, a significant change was not expected (33). Comparison of the number of secondary follicles in the studied groups showed that healthy rats have the highest number and PCOs animals have the lowest number. After 28 days of SA administration, a recovery process has been observed. The experimental groups had the highest number of secondary follicles in a dose-dependent manner. Based on previous studies, the role of SA on estrogen is probably due to the presence of quercetin, daucosterol, and sircinol (23, 24). Estrogen surge is secondary to LH surge. High levels of LH cause follicles to growth and enter the antral phase. Although no study has examined the ovarian tissue of the specimens, this finding can be inferred from the findings of previous studies (increased LH and estrogen levels).

Graph (or tertiary) follicles had the highest number in healthy groups and the lowest number in PCOs groups. Graph follicles in the PCOs groups were so small that no follicle graph was observed in this group for 89 days. On the other hand, although SA extract was relatively dose-dependent in improving the ovarian status, it was significantly lower than normal for graph follicles, unlike other follicles. Therefore, it appears that the most stable PCOs-induced abnormality in follicles clearly affects the number of graph follicles.

We do not expect the atresia follicles to be significantly higher than normal in PCOS. This is because the follicles often become atretic at the end of the second phase (early antral phase). On the other hand, in PCOs, the ovary becomes polycystic because the immature follicles are not atretic, and this appearance does not appear in normal people due to the atresia of follicles that are not supposed to participate in fertilization. It is also normal to observe atresia follicles at the end or beginning of the next cycle. Although no study has been done on the effect of SA on ovarian tissue, this finding is inconsistent with previous knowledge about this syndrome.

As at the beginning of the article, PCOs causes infertility. Therefore, one of the limitations of this article is the inability to measure the effects of SA on the fertility of mated animals.

## 5. Conclusion

The results of this study show that SA can positively affect the improvement PCOs. This effect can be achieved by regulating the level of sex hormones as well as correcting follicular abnormalities in ovarian tissue. Therefore, it can be said that it is possible that SA can be used as a complementary drug in PCOs.

##  Data availability

Data supporting the findings of this study are available upon reasonable request from the corresponding author.

##  Author contributions

A.Z and H.K.J proposed the presented idea. H.A, H.K.J, A.Z, H.H, and N.P performed the experiments. H.A, B.E, and H.K.J, wrote the manuscript with support from other authors. The manuscript has been read and approved by all authors.

##  Conflict of Interest

The authors declare that there is no conflict of interest.
